# Agro-Morphological Characterization of Lentil Germplasm of Indian National Genebank and Development of a Core Set for Efficient Utilization in Lentil Improvement Programs

**DOI:** 10.3389/fpls.2021.751429

**Published:** 2022-01-27

**Authors:** Kuldeep Tripathi, Jyoti Kumari, Padmavati G. Gore, Dwijesh C. Mishra, Amit Kumar Singh, Gyan P. Mishra, C. Gayacharan, H. K. Dikshit, Neeta Singh, D. P. Semwal, Reena Mehra, Rakesh Bhardwaj, Ruchi Bansal, J. C. Rana, Ashok Kumar, Veena Gupta, Kuldeep Singh, Ashutosh Sarker

**Affiliations:** ^1^Indian Council of Agricultural Research-National Bureau of Plant Genetic Resources, New Delhi, India; ^2^Indian Council of Agricultural Research-Indian Agricultural Statistics Research Institute, New Delhi, India; ^3^Division of Genetics, Indian Council of Agricultural Research-Indian Agricultural Research Institute, New Delhi, India; ^4^International Center for Agricultural Research in Dry Areas-Food Legumes Research Platform, Amlaha, India; ^5^The Alliance of Bioversity International and CIAT, National Agricultural Science Complex, New Delhi, India

**Keywords:** *Lens culinaris*, characterization, core-set, genetic diversity, trait-specific germplasm

## Abstract

Lentil (*Lens culinaris* Medik.) is one of the major cool-season pulse crops worldwide. Its increasing demand as a staple pulse has led to the unlocking of diverse germplasm collections conserved in the genebanks to develop its superior varieties. The Indian National Genebank, housed at the Indian Council of Agricultural Research (ICAR)-National Bureau of Plant Genetic Resources, New Delhi, India, currently has 2,324 accessions comprising 1,796 indigenous and 528 exotic collections. This study was conducted to unveil the potential of lentil germplasm by assessing its agro-morphological characteristics and diversity, identifying trait-specific germplasm, and developing a core set. The complete germplasm set was characterized for two years, i.e., 2017–2018 and 2018–2019, and data were recorded on 26 agro-morphological traits. High phenotypic variability was observed for nine quantitative and 17 qualitative traits. A core set comprising 170 accessions (137 Indian and 33 exotic) was derived based on the characterization data as well as geographical origin using a heuristic method and PowerCore software. This core set was found to be sufficiently diverse and representative of the entire collection based on the comparison made using Shannon–Weaver diversity indices and χ^2^ test. These results were further validated by summary statistics. The core set displayed high genetic diversity as evident from a higher coefficient of variance in comparison to the entire set for individual traits and overall Shannon–Weaver diversity indices (entire: 1.054; core: 1.361). In addition, the total variation explained by the first three principal components was higher in the core set (70.69%) than in the entire collection (68.03%). Further, the conservation of pairwise correlation values among descriptors in the entire and core set reflected the maintenance of the structure of the whole set. Based on the results, this core set is believed to represent the entire collection, completely. Therefore, it constitutes a potential set of germplasm that can be used in the genetic enhancement of lentils.

## Introduction

Lentil (*Lens culinaris* Medik.; 2n = 2 × = 14) is a diploid, self- pollinating, and cool-season pulse that is grown worldwide (Kumar and Gupta, 2020). It is regarded as one of the founder crops of neolithic agriculture (Zohary and Hopf, 1973). In addition, the global productivity of lentils has increased from an average yield of 806 kg ha^–1^ to 1194.6 kg ha^–1^ during the last two decades (FAOSTAT, 2021). In India, lentil ranks as the second-most important winter pulse crop after chickpea. In 2019, 1.23 million tons of lentil was produced in India, with a mean productivity of average yield of 901 kg ha^–1^ (FAOSTAT, 2021). However, India’s current yield of lentils is considerably lower than that of several other countries because of the poor yield of cultivars.

Poor seedling vigor, low biomass, delicate stem, low harvest index, lodging, less conversion of flower to the pod, and climate-induced stresses are the primary yield-reducing factors in lentils (Erskine et al., 2009). In addition, the narrow genetic base or limited parentage of modern varieties has emerged as a major concern for lentil improvement. Consequently, the potential genetic gains in lentil productivity could not be achieved. Genebanks are the source of genes exhibiting valuable traits that can be utilized not only to develop superior varieties but also those with tolerance to stresses induced due to changing climate (Díez et al., 2018). Therefore, it is essential to search genebanks and identify novel germplasm for its use in lentil-breeding programs to enhance cultivar productivity and resilience to climate change. The efficient use of genetic diversity in the varietal development program is an effective method to enhance yield gains and cope with the emerging climate-induced stresses.

To identify novel accessions corresponding to the genes with desired traits, it is prudent to characterize the germplasm collections conserved in the genebanks. However, the characterization of extensive collections by plant breeders is time- and resource-consuming, genebank curators have developed a concept of a core set from entire collections that are characterized at once to identify the targeted germplasm and use it efficiently (Frankel and Brown, 1984; Brown, 1989; van Hintum et al., 2000). The core set refers to a minimum set of germplasm that captures the entire range of genetic variability of any crop, with minimum repetitiveness. Being smaller in size and diverse in nature, the core set can be efficiently used as a kickoff point to enhance genetic gains, including the use of phenomics and genomics tools in less time. Globally, legume germplasm curators have developed core sets in soybean (Oliveira et al., 2010), cowpea (Mahalakshmi et al., 2007), pigeonpea (Reddy et al., 2005), groundnut (Upadhyaya et al., 2003), chickpea (Upadhyaya et al., 2001), lentil (Simon and Hannan, 1995; Tullu et al., 2001), and lablab bean (Vaijayanthi et al., 2015) to enhance the germplasm use. The Indian Council of Agricultural Research-National Bureau of Plant Genetic Resources (ICAR-NBPGR) has developed core sets for Indian mustard (Nanjundan et al., 2021), wheat (Phogat et al., 2020), chickpea (Archak et al., 2016), wild lens (Singh et al., 2014), brinjal (Gangopadhyay et al., 2010), and mungbean (Bisht et al., 1998). Therefore, the aim of the present study was to develop a core set of cultivated lentil germplasm conserved in Indian national genebank based on agro-morphological characterization and diversity indices for accelerating utilization of germplasm in lentil breeding programs.

## Materials and Methods

### Plant Material

**FIGURE 1:**
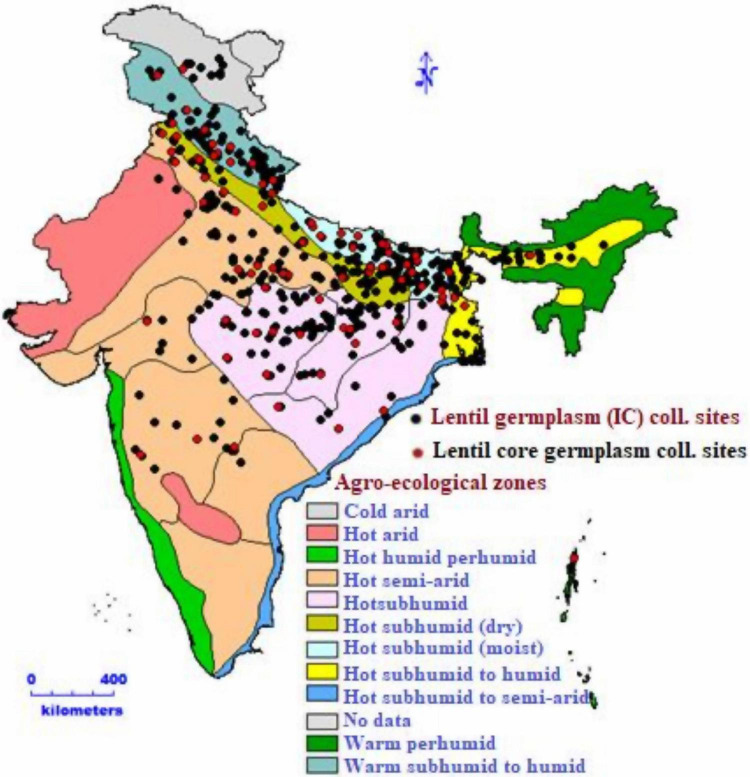
Indigenous lentil collection sites of national genebank from different agro-ecological zones.

**FIGURE 2:**
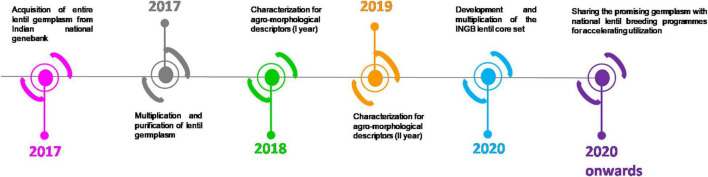
Timeline of the present investigation from 2017 until 2020.

The study material consisted of 2,324 cultivated lentil germplasm accessions comprising 1,796 indigenous collections (ICs) and 528 exotic collections (ECs). The indigenous accessions were collected from different lentil-growing areas in India after exploring several crops from 1976 to 2017. Geo-coordinates of each collection site were mapped using geographic information system (GIS) tools (Figure 1). Geo-referenced maps were prepared with WGS84 datum and geographic projection system using GIS tools (Semwal et al., 2014). The germplasm was characterized during the winter season of 2017–2018 and 2018–2019 at Research Farm of ICAR-NBPGR located at a latitude of 28°38′N and longitude of 77°10′ E and an altitude of 228.61 m above the mean sea level. The soil of the location was sandy loam type having an optimal pH range. Lentil germplasm accessions were sown under natural field conditions. The recommended package of practices for growing lentil was followed. To meet the crop’s balanced nutrient demand, 20 kg ha^–1^ nitrogen and 40 kg ha^–1^ phosphorus was applied as a basal dose before sowing. One presowing irrigation was given to ensure proper germination in the field, while irrigation was also given at the pod formation stage. The seeds were treated with a mixure of thiram (2g) and carbendazim (1 g) per kg of seed. Manual weeding was completed at 25–30 and 45–50 days after sowing. The experimental design consisted of an augmented block (Federer and Raghavarao, 1975) with four popular checks, namely, DPL62, IPL316, IPL526, and L4717. The experiment was replicated in 24 blocks. A hundred accessions were sown per block uniformly in 23 blocks, whereas, the 24^th^ block was incomplete with 24 accessions. The accessions were sown in paired rows of two m length, with a row-to-row distance of 30 cm. The detailed timeline of the experiment is shown in Figure 2.

### Agro-Morphological Descriptors Used in the Experiment

Twenty-six agro-morphological descriptors (nine quantitative and 17 qualitative) were selected for phenotypic characterization using descriptor lists of lentil (IBPGR, 1985; Mahajan et al., 2000) (Table 1). The qualitative characters were (1) early plant vigor (EPV), (2) seedling stem pigmentation (SSP), (3) growth habit (GH), (4) leaf color (LC), (5) leaf pubescence (LP), (6) leaflet size (LS), (7) flower ground color (FGC), (8) tendril length (TL), (9) biomass score (BS), (10) lodging score (LoD), (11) pod pigmentation (PP), (12) pod dehiscence (PD), (13) pod shedding (PS), (14) seed shape (SS), (15) seed coat color (SCC), (16) pattern of seed testa (PST), and (17) cotyledon color (CC). The quantitative traits included (1) days to 50% flowering (DF), (2) days to 80% maturity (DM), (3) plant height (PH), (4) number secondary branches per plant (SBP), (5) seed diameter (SD), (6) seed thickness (ST), (7) pods per plant (PPP), (8) seeds per pod (SPP), and (9) 100-seed weight (SW).

**TABLE 1:** Descriptors used for agro-morphological characterization of lentil germplasm of Indian national genebank.

S. No.	Descriptors	Code	Stage of observation	Descriptor state
**Qualitative traits**				
1	Early plant vigor	EPV	25 DAS	Poor (1), Good (2), Very Good (3)
2	Seeding stem pigmentation	SSP	45DAS	Absent (0), Present (1)
3	Growth habit	GH	50% flowering	Erect (1), Semi-erect (3), Horizontal (5)
4	Leaf color	LC	50% flowering	Green (1), Light green(2), Pigmented (3)
5	Leaf pubescence	LP	50% flowering	Absent (0), Slight (3), Medium (5), Dense (7)
6	Leaflet size	LS	On lower flowering nodes	Small (3), Medium (5), Large (7)
7	Flower ground color	FGC	Open flower in the morning	White (1), Yellow (2), Red (3), Purple (4)
8	Tendril length	TL	Pod filling	Rudimentary (1) Prominent (2)
9	Biomass score	BS	Mid pod filling	Low (3), Medium (5), High (7)
10	Lodging score	LoD	Mid pod filling	Absent (0), Present (1)
11	Pod pigmentation	PP	Near to maturity	Absent (0), Present (1)
12	Pod dehiscence	PD	Scored a week after maturity	Absent (0), Low (3), Medium (5), High (7)
13	Pod shedding	PS	Scored a week after maturity	None (0), Low (3), Medium (5), High (7)
14	Seed shape	SS	Post-harvest	Flattened (1), Globose (2)
15	Seed coat color	SCC	Post-harvest	Yellow (1), Green (2), Brown (3), Pink (4), Gray (5), Black (6)
16	Pattern of seed testa	PST	Post-harvest	Absent (0), Dotted (1), Spotted (2), Marbled (3), Complex (4)
17	Cotyledon color	CC	Post-harvest	Yellow (1), Orange (2), Green (3)
**Quantitative traits**				
18	Seed diameter (mm)	SD	Post-harvest	Average of five replications
19	Seed thickness (mm)	ST	Post-harvest	Average of five replications
20	Days to 50% flowering	DF	50% flowering	Plot basis
21	Secondary branches per plant	SBP	Maturity	Average of five replications
22	Plant height (cm)	PH	Late pod filling stage	Average of five replications
23	Pods per plant	PPP	Maturity	Average of five replications
24	Seeds per pod	SP	Maturity	Average of five replications
25	Days to 80% maturity	DM	80% maturity	Plot basis
26	100-seed weight (g)	SW	Post-harvest	Average of five replications

### Development and Validation of Core Collections

A statistical software, PowerCore, was used to constitute a core collection based on the characterization data generated using 26 different descriptor traits (Kim et al., 2007). Continuous variables were classified into different categories based on Sturges’ rule (Sturges, 1926) (K = 1 + log_2_n), where *n* and *K* mean the number of observed accessions and number of classes, respectively. The number of classes can be modified. The modified heuristic algorithm was implemented to identify and select accessions of core collections using the strategy of advanced maximization or allelic richness. Subsets created using PowerCore represented all observation classes with the least allelic redundancy, ultimately ensuring a highly reproducible entry list. Core sets were extracted with different combinations of variables to assess their efficacy using PowerCore and using qualitative traits, quantitative traits, a combination of quantitative and qualitative traits, and a combination of quantitative and qualitative traits and passport data. Another method, known as principal component score strategy (PCSS), was used to extract the core (Noirot et al., 1996). This method uses principal component analysis (PCA) to eliminate collinearity between variables and sample individuals based on their cumulative relative contribution. The Newman–Keuls procedure was followed to compare the means of the entire collection and developed core sets (Newman, 1939; Keuls, 1952) for descriptor traits. The similarity of distribution frequencies in the core and entire sets was evaluated using the χ^2^ test. The Shannon–Weaver diversity index (SDI) was used to compare the representativeness of the whole set and the germplasm selected as core entries.

### Data Analysis

The data recorded for nine quantitative traits were first analyzed separately for each year, after which a combined analysis was performed using the general linear model (PROC GLM) method of SAS 9.3 for augmented design. The accessions were considered random and year as a fixed effect. Levene’s test was performed to assess the homogeneity of error variances before performing the combined analysis (Levene, 1960). Descriptive statistics were estimated for the complete set and the core collections separately. Frequency distribution for different classes of qualitative traits was obtained using Excel, and histograms for quantitative traits were drawn using IBM statistical package for the social sciences (SPSS) statistics (version 20.0). Variability in descriptor traits between ICs and ECs was compared using boxplots developed using Statistical Analysis Software-JMP 14 software. Hierarchical cluster analysis was performed using the Euclidean distance matrix following Ward’s minimum variance method for accession number grouping and estimating genetic relationships. Core sets were extracted using PowerCore and the PCS strategy. The principles laid by Oliveira et al. (2010) were used to calculate the range retention percentage. In addition, the distribution of homogeneity for each of the descriptor traits was analyzed using the χ^2^ test. For the quantitative traits, classes were formed based on Sturges’ formula. The observed number of accessions in the core set in each class was determined and was tested against the expected number of accessions using the χ^2^ test. Further, the SDI (H’) was computed as a measure of phenotypic variability using the phenotypic frequencies of quantitative and qualitative traits (Shannon and Weaver, 1949) in the entire and different core sets extracted using the following formula.


$$H^{\prime }=-\sum_{i=1}^{n}\mathrm{pi}.\ln\mathrm{pi}$$

where p_i_ is the proportion of germplasm accessions in the i^th^ class of an n-class characteristic and n is the number of phenotypic categories for a character. Phenotypic correlation coefficients (*r*) among descriptors in the core collection (Core^d^) and entire set were estimated. Moreover, the contribution of different descriptor traits to multivariate polymorphism and conservation of contribution in the core collection were assessed based on PCA. The SAS 9.3 program was used to estimate the correlation and PCA (SAS Institute, 2012).

## Results

### Agro-Morphological Characterization of Lentil Germplasm

**FIGURE 3:**
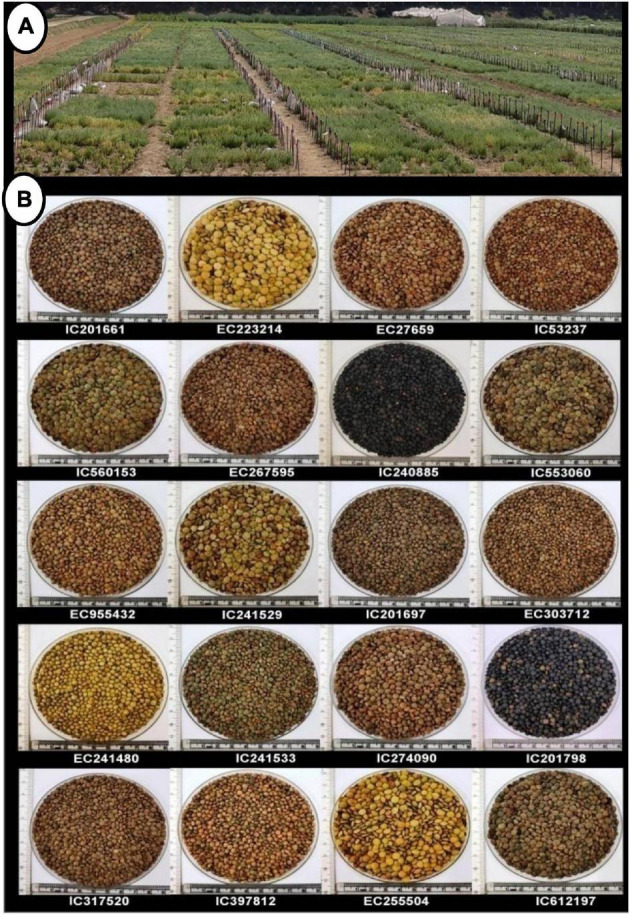
**(A)** Field view of agro-morphological characterization of lentil germplasm and **(B)** seed coat diversity.

The actual germplasm collection sites of lentils in India (Figure 1) were presented using geo-referenced map. These maps revealed that most of the lentil germplasm were collected from Uttar Pradesh, Bihar, Uttarakhand, and Madhya Pradesh. Geo-referenced map showed that lentils were collected from Western Himalayas (Kashmir, Himachal Pradesh, and Uttarakhand) to Eastern Himalayas (Assam and Arunachal Pradesh). It was collected from high-altitude areas in north (Leh) to low-lying areas of eastern part of India (West Bengal). Collections from dry areas of Rajasthan to wet areas of North-east Himalayas indicated that India has sufficient diversity in its collections. Indian lentil collections showed the unique distribution pattern having representation from four global biodiversity hotspots, the Himalayas, the Western Ghats, the Indo-Burma region, and the Sundaland (Includes Nicobar group of Islands). All 2,324 accessions and four checks were characterized under the agro-climatic region in the north-western Indian conditions for two consecutive years (2017–2018 and 2018–2019). The experimental field view and seed coat color diversity are presented in Figure 3.

### Characterization Using Qualitative Descriptor Traits

**FIGURE 4:**
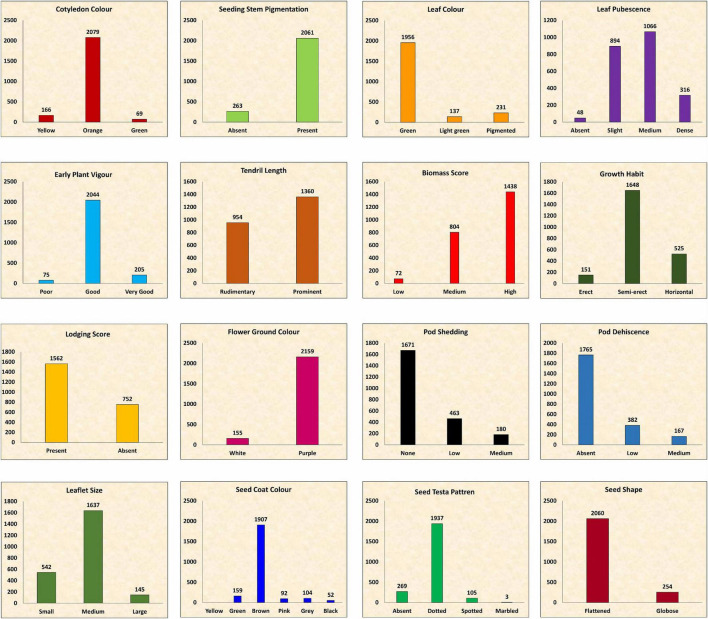
Frequency distribution of entire lentil germplasm for qualitative traits.

The frequency distribution of the entire set was not normal and varied among traits. In certain cases, a particular trait was predominant, whereas it was dispersed in other cases (Figure 4). The majority of the accessions (2,061) showed purple pigmentation on the stem at the seedling stage, whereas leaf color was recorded as green. A slight to medium leaf pubescence was prominent, whereas 48 accessions were glabrous. EPV of most of the accessions (2,044) was good, whereas 275 accessions were highly vigorous. Prominent tendril was recorded in 1,360 accessions, whereas 954 accessions showed rudimentary tendril. High biomass was observed at the mid pod filling stage in 1,438 accessions, whereas 72 accessions showed low biomass. Semi-erect plant growth was more prominent (1,648 accessions), and horizontal growth was recorded in 525 accessions, whereas the growth in 151 accessions was erect. Around 67% of the accessions showed lodging at the time of pod filling. The maximum number of accessions (2,159) were of purple flower ground color. After a week of maturity, medium pod shedding was recorded in 180 accessions, whereas most of the accessions (1,671) showed no pod shedding. Pod dehiscence was absent in 1,765 accessions, whereas low to medium pod dehiscence was noted in 549 accessions. Pod pigmentation was absent in the majority of the accessions, whereas brown seed coat color and dotted testa pattern were prominent. A flattened seed shape was observed in 2,060 accessions, whereas 254 accessions showed the globose seed shape. There was substantial variation in the cotyledon color with a maximum of orange type.

### Agronomic Evaluation Using Quantitative Traits

**FIGURE 5:**
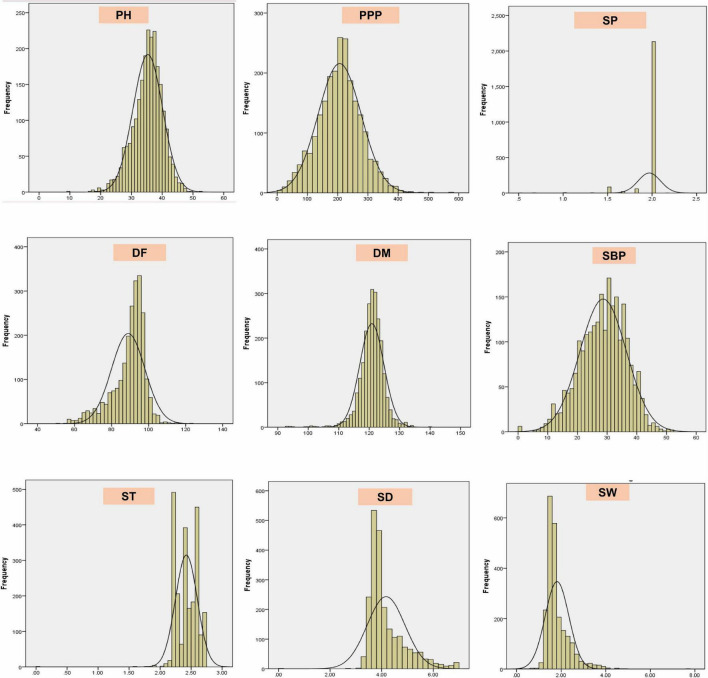
Frequency distribution plots showing the variability of nine quantitative traits in the entire lentil germplasm conserved in Indian national genebank.

**FIGURE 6:**
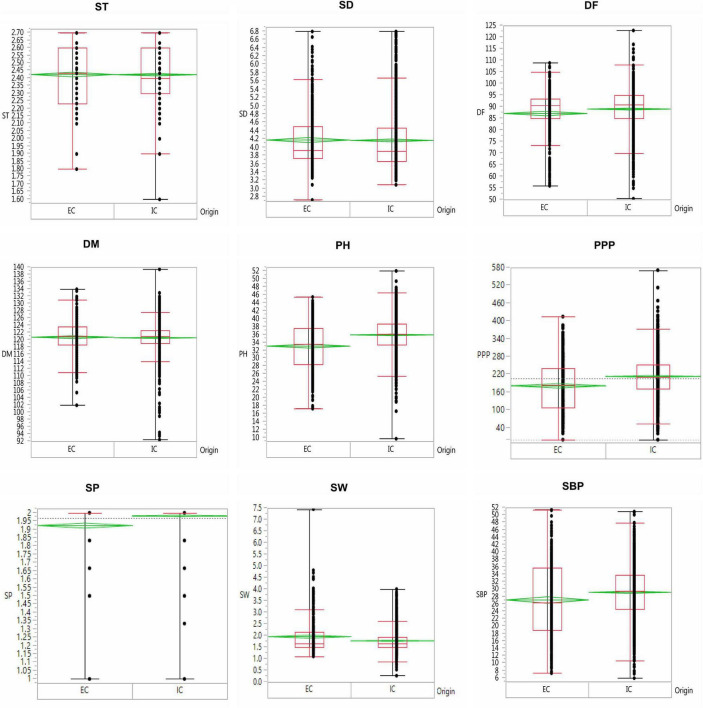
Boxplot depicting the variability of traits in the indigenous collection (IC) and exotic collection (EC) of the entire lentil germplasm conserved in Indian national genebank.

The results revealed significant variations as evident from the mean, range, and coefficient of variations (Supplementary Table 1 and Figure 5). The days to 50% flowering and maturity ranged from 51 to 123 and 93 to 140 days, respectively, whereas 100-seed weight varied from 0.75 to 7.6 g. The average seed thickness and seed diameters were 2.42 and 4.18 cm, respectively. The mean plant height and the number of secondary branches were 35.34 and 28.64 cm, respectively. The coefficient of variation ranged from low (<10.17%; days to flowering, seed thickness, days to maturity, and number of seeds per pod) to high (>17.34%; the number of secondary branches, 100-seed weight, number of pods per plant, and seed diameter) for key descriptor traits. In addition, substantial variation in the range for the number of pods per plant, number of secondary branches per plant, 100-seed weight, and earliness was observed. The least variation was observed in the number of seeds per pod, with the majority of the accessions with two seeds per pod in studied accessions. ICs showed earliness, small seeds, and low biomass as compared to the ECs. The days to 50% flowering, days to 80% maturity, and seeds per pod were negatively skewed, indicating that a majority of the accessions had higher mean values for these traits and days to 80% maturity and seeds per pod showed high kurtosis values with a relatively higher peak. Boxplot analysis revealed a comparison of trait distribution between exotic and indigenous accessions. The average performance for plant height, days to 50% flowering, pods per plant, and number of secondary branches was lower in ECs than in ICs. However, the mean value of seed weight was higher in ECs than in ICs (Figure 6).

### Clustering of Lentil Germplasm Accessions

**FIGURE 7:**
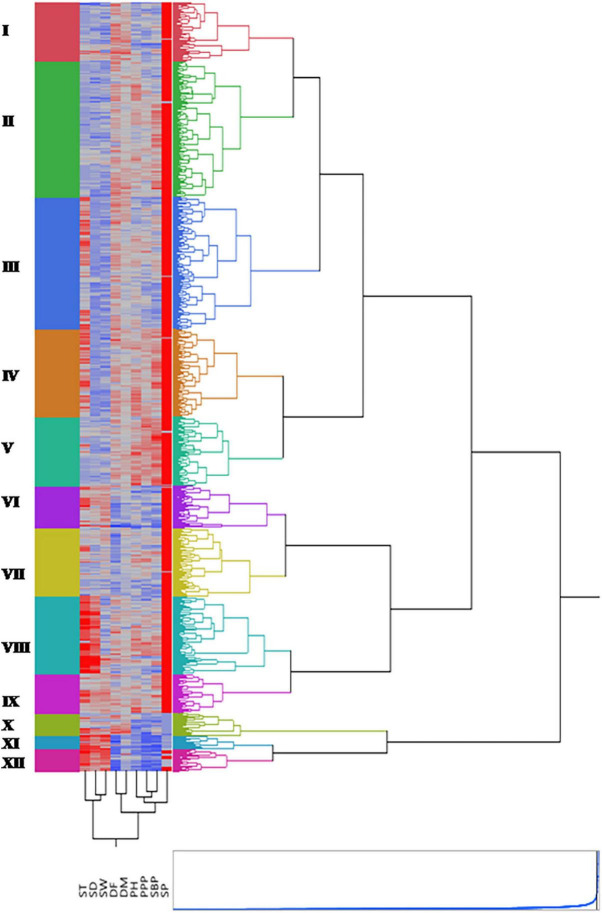
Dendrogram along with heatmap of entire germplasm generated by performing hierarchical cluster analysis. Twelve clusters are represented by different colors. The two-dimensional heatmap is represented by columns and rows. Each column represents different quantitative trait and each row an accession. The higher the trait value, the brighter is the red and similarly, lower the trait value, brighter is the blue color, respectively.

Grouping several accessions into certain homogenous clusters assists in selecting diverse parents. This allowed the accurate comparison among all probable pairs of individuals and brought together gene constellations, yielding desired progenies through crossing between different parents. Hierarchical cluster analysis with Ward’s method of minimum variance was applied to assess genetic diversity. Based on nine quantitative traits, phenotypic relationships among lentil accessions were determined using Euclidean distances. The Indian national gene bank (INGB) lentil accessions were grouped into 12 clusters with a varied number of accessions in each cluster (Figure 7 and Supplementary Table 2). A list of core set accessions with cluster number is given in Supplementary Table 3. The cluster also showed heatmap distribution of accessions, showing trait variability in different clusters represented by different colors. Cluster II was the largest with 393 accessions followed by Clusters III and IV with 382 and 253 accessions, respectively. Cluster II grouped 320 ICs and 73 ECs accessions; whereas Cluster XI was the smallest with only 38 accessions (24 ECs and 14 ICs). The cluster mean values of studied traits showed that Clusters XI and XII included extra-early accessions, such as IC241529, IC241531, and IC241532, whereas early to medium maturing accessions were grouped into Clusters VI and VII. Late flowering accessions such as IC73690, IC73692, IC381127, and IC398044 grouped in cluster I. Proportionately, exotic accessions were relatively dominant in Clusters X, XI, and XII, and indigenous were dominant in Clusters II to IX. Cluster XII comprised all exotic accessions from Syria. Cluster V contained accessions having very tall plant types (IC329110, IC398793, and IC59038, a high number of pods per plant (IC78387 and IC78398), and a high number of secondary branches (IC148333, IC16453). The accessions such as EC223214 and IC241543 grouped in cluster XII had highest mean value for seed weight (3.239 g) whereas cluster III had accessions with mean value of 1.523 g.

### Identification of Trait-Specific Germplasm

**FIGURE 8:**
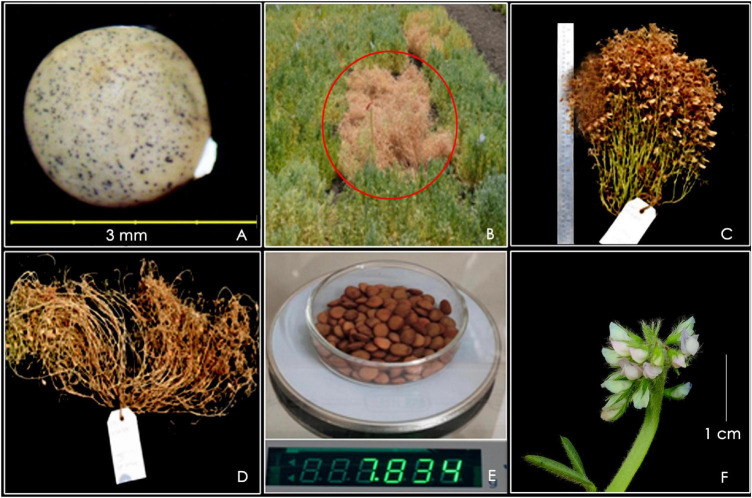
Trait-specific lentil germplasm, **(A)** IC317520: unique seed morphotype with an intact funiculus; **(B)** IC241532: early maturing accession (circled in red) relative to surrounding germplasm; **(C)** EC267615: suitable to mechanical harvesting; **(D)** IC199461: high number of secondary branches; **(E)** EC499760: bold seeded type with high 100-seed weight; **(F)** IC241473: multiflowering germplasm.

Potential accession numbers for unique traits were identified from characterization datasets and validated over the years and locations at NBPGR, New Delhi, The International Center for Agricultural Research in the Dry Areas (ICARDA), Amlaha, and NBPGR, Regional Station, Ranchi. Some of them are depicted in Figure 8. The accession IC317520 was identified with a unique seed morphotype having extended funiculus. A total of 50% of plants with accessions such as IC241532 and IC241529 exhibited very early flowering (51 days) relative to all checks used in the study. The accession EC267615 displayed a novel type of erect plant architecture, indicating its suitability for mechanical harvesting. Pod setting in this genotype was observed at more than 15 cm above the ground. The accessions IC199461 and IC201716 had a high number of secondary branches (>50) and pods per plant (>400) relative to all checks used in the study. The accession EC499760 was of bold seeded type (100-seed weight, 7.1–7.83 g) and was imported from the United States of America (USA). A novel and unique multiflower (MF) germplasm accession, IC241473, was identified the first time that formed up to 16 flowers per peduncle (FPP) at multiple flowering nodes. In addition, this accession showed fasciation of the main stem.

### Development and Validation of Core Set

The core sets were extracted using combinations of different datasets using the PowerCore software. Additionally, the PCSS was used to develop the core sets. These core sets were designated as Core^a^, Core^b^, Core^c^, Core^d^, and Core^e^ when generated using qualitative traits, quantitative traits, a combination of both traits, a combination of both traits and passport data, and PCSS. A total of 170 accessions were selected based on the trio of qualitative and quantitative traits and passport data using PowerCore (Table 2 and Supplementary Table 3). A total of 67 accessions were selected when the data of only qualitative variables were used, whereas 71 accessions and 79 accessions were selected, respectively, when data of only quantitative characteristics and both variables were used for the analysis. Accessions with the maximum relative contribution corresponding to 10% representation were used as the criteria to select the core; thus, 221 accessions were selected based on the PCS strategy. Relative SDI was estimated for both quantitative and qualitative traits in entire lentil accessions and in core sets developed using a different strategy that revealed adequate diversity. The lentil germplasm was more diverse (*H*’ > 0.5) for all descriptors, except for EPV, SSP, FGC, PP, SS, CC, and SP. The average SDI for quantitative traits was higher than qualitative traits. The SDIs were consistently higher in the core sets derived from different datasets than in the entire set, signifying an enhanced representation of the available diversity in each core set. PowerCore yielded a better representation of diversity than PCSS. The average SDIs for all traits revealed that Core^d^ (1.361, 170 accessions) was more diverse, followed by Core^b^ (1.340, 71 accessions) and Core^a^ (1.302, 67 accessions) (Table 2).

**TABLE 2:** Comparison of the Shannon–Weaver diversity index (SDI) of the entire lentil collections to lentil core sets derived from different datasets and strategies.

Traits	Entire	Core^a^	Core^b^	Core^c^	Core^d^	Core^e^
Leaflet size	0.744	0.812	0.780	0.805	0.828	0.682
Growth habit	0.770	1.110	0.959	0.928	1.080	0.082
Early plant vigor	0.441	0.739	0.783	0.756	0.817	0.302
Seeding stem pigmentation	0.357	0.678	0.600	0.588	0.671	0.309
Leaf color	0.548	0.939	0.872	0.856	0.916	0.258
Leaf pubescence	1.069	1.113	1.119	1.115	1.099	1.148
Flower ground color	0.256	0.619	0.525	0.458	0.597	0.404
Tendril length	0.689	0.731	0.696	0.718	0.749	0.643
Pod pigmentation	0.016	0.121	0.085	0.082	0.140	0.004
Pod dehiscence	0.706	0.662	0.639	0.786	0.695	0.617
Pod shedding	0.770	0.799	0.689	0.871	0.809	0.622
Seed shape	0.378	0.381	0.395	0.504	0.387	0.264
Seed coat color	0.670	1.040	0.871	0.927	1.006	0.775
Pattern of seed testa	0.571	0.918	0.849	0.759	0.895	0.650
Biomass score	0.854	1.177	1.121	1.071	1.161	0.908
Lodging score	0.632	0.668	0.682	0.677	0.688	0.651
Cotyledon color	0.288	0.585	0.628	0.441	0.606	0.544
Seed thickness	1.667	1.643	1.909	1.811	1.942	1.684
Seed diameter	1.570	1.656	1.841	1.729	1.889	1.749
Days to 50% flowering	1.691	2.332	2.259	2.060	2.230	1.901
Days to 80% maturity	1.358	1.664	1.993	1.708	1.928	1.609
Plant height	1.726	2.118	2.215	2.035	2.180	1.780
Pods per plant	1.825	1.886	1.953	1.904	1.973	2.051
Seeds per pod	0.375	0.873	1.056	0.714	1.003	0.599
100-seed weight	1.466	2.124	2.128	1.855	2.126	1.832
No. of secondary branches	2.121	2.201	2.266	2.267	2.263	2.120
No. of accessions	2,324	67	71	79	**170**	221
Average SDI (Quantitative traits)	1.553	1.833	1.958	1.787	**1.948**	1.703
Average SDI (Qualitative traits)	0.574	0.770	0.723	0.726	**0.773**	0.521
Average SDI (Overall)	1.054	1.302	1.340	1.257	**1.361**	1.112

*Core^a^, Core^b^, Core^c^, Core^d^, and Core^e^ referred to core sets generated using qualitative traits, quantitative traits, a combination of both traits, a combination of both traits and passport data, and principal component score strategy (PCSS). Bold values indicating the selected core set with maximum diversity (highest SDI value in Core^d^).*

Qualitative traits in the INGB collections were represented in the core sets developed by all strategies, except the core developed by quantitative method and PCSS strategy, indicating that the Core^a^, Core^c^, and Core^d^ captured the allelic richness of the entire INGB collections. The range of quantitative descriptors was classified into 12 groups for frequency distribution and χ^2^ analysis based on PowerCore. Frequency distribution analysis indicated the homogeneity of distribution of several traits by Core^a^(10), Core^b^(13), Core^c^(11), Core^d^ (18), and Core^e^(9) (Table 3). Thus, the core set developed by combining qualitative and quantitative traits and passport data well-represented the structure of the entire lentil germplasm. The results showed that the variability existing in the entire set was well-symbolized in the core collection. Differences in the means of the entire collection and Core^d^ were insignificant for quantitative descriptors, except days to flowering and seed weight. In addition, the range retention percentage was 100, capturing a complete range of diversity, including all extremes (Table 4).

**TABLE 3:** Chi-square test for comparison of frequency distribution for descriptor traits studied in lentil core sets derived from different datasets and strategies.

Traits	Core^a^	Core^b^	Core^c^	Core^d^	Core^e^
Leaflet size	2.209^ns^	3.009^ns^	2.118^ns^	2.629^ns^	81.484*
Growth habit	12.281*	12.680^ns^	12.195*	7.216*	743.761*
Early plant vigor	24.694*	20.834*	29.412*	22.345*	108.249*
Seeding stem pigmentation	11.724^ns^	12.546^ns^	12.518^ns^	14.739^ns^	1538.154*
Leaf color	15.716*	13.723*	18.720*	19.387*	502.182*
Leaf pubescence	4.385^ns^	4.359^ns^	4.552^ns^	2.403^ns^	4.359^ns^
Flower ground color	10.897*	7.337^ns^	10.941*	8.043*	9.627*
Tendril length	1.379^ns^	0.233*^ns^*	1.198^ns^	1.641^ns^	4.481*
Pod pigmentation	1.065*	1.063^ns^	1.406^ns^	1.061^ns^	0.006^ns^
Pod dehiscence	4.156^ns^	4.195^ns^	1.782*^ns^*	0.562^ns^	2.207^ns^
Pod shedding	2.099^ns^	5.461*	2.172^ns^	3.698*	8.700*
Seed shape	3.694^ns^	6.628^ns^	6.622^ns^	3.541^ns^	6.447^ns^
Seed coat color	11.690^ns^	15.299^ns^	15.238^ns^	13.443^ns^	113.237*
Pattern of seed testa	15.958*	14.860^ns^	19.354*	8.335^ns^	25.439^ns^
Biomass score	11.031^ns^	12.283^ns^	16.299^ns^	15.155^ns^	2.187^ns^
Lodging score	26.989^ns^	27.143^ns^	35.654^ns^	23.490^ns^	0.835^ns^
Cotyledon color	10.287*	14.811*	14.292*	5.529^ns^	27.990*
Seed thickness	235.628*	135.150*	94.694*	20.108^ns^	146.220*
Seed diameter	69.870*	92.813*	81.421*	18.659^ns^	229.906*
Days to 50% flowering	96.521*	77.337*	116.731*	34.433*	24.360*
Days to 80% maturity	52.890*	36.112*	39.905*	19.101^ns^	24.250*
Plant height	24.272*	34.940*	36.027*	18.916^ns^	17.800^ns^
Pods per plant	58.418*	81.329*	53.987*	33.097^ns^	55.711*
Seeds per pod	9.737*	12.215*	12.572*	19.969*	9.888*
100-seed weight	38.426*	47.352*	52.075*	77.273*	74.607*
No. of secondary branches	41.033*	53.699*	40.724*	28.447^ns^	26.303^ns^

Core^a^, Core^b^, Core^c^, Core^d^, and Core^e^ referred to core sets generated using qualitative traits, quantitative traits, a combination of both traits, a combination of both traits and passport data, and principal component score strategy (PCSS). *P < 0.05.

**TABLE 4:** Mean, range, and CV in entire and core set of lentil germplasm.

Traits	Mean			Minimum	Maximum	Minimum	Maximum	CV (%)	
							
	Entire	Core^d^	Significance level	Entire		Core^d^		Entire	Core^d^
ST	2.42 ± 0.01	2.41 ± 0.01	Ns	1.60	2.70	1.60	2.70	6.96	7.68
SD	4.18 ± 0.02	4.19 ± 0.06	Ns	2.73	6.80	2.73	6.80	17.41	18.38
DF	88.96 ± 0.19	85.43 ± 1.02	*	51.00	123.00	51.00	123.00	10.17	15.41
DM	120.88 ± 0.08	119.50 ± 0.51	Ns	93.00	140.00	93.00	140.00	3.28	5.55
PH	35.34 ± 0.10	34.04 ± 0.51	Ns	9.70	52.13	9.70	52.13	13.64	19.41
PP	206.38 ± 7.48	183.48 ± 6.73	Ns	20.83	571.33	20.83	571.33	34.56	47.69
SP	1.97 ± 0.01	1.92 ± 0.02	Ns	1.00	2.00	1.00	2.00	6.38	12.47
SW	1.91 ± 0.01	1.99 ± 0.06	**	0.75	7.6	0.75	7.6	28.50	39.50
SBP	28.67 ± 0.16	26.91 ± 0.73	Ns	6.00	51.50	6.00	51.50	27.33	35.16

*ST, seed thickness; SD, seed diameter; DF, days to 50% flowering; SBP, secondary branches per plant; PH, plant height; PP, pods per plant; SP, seeds per pod; DM, days to 80% maturity; SW, 100-seed weight.*

*Significant at 5% level.

**Significant at 1% level.

### Correlation and Principal Component Analysis

**FIGURE 9:**
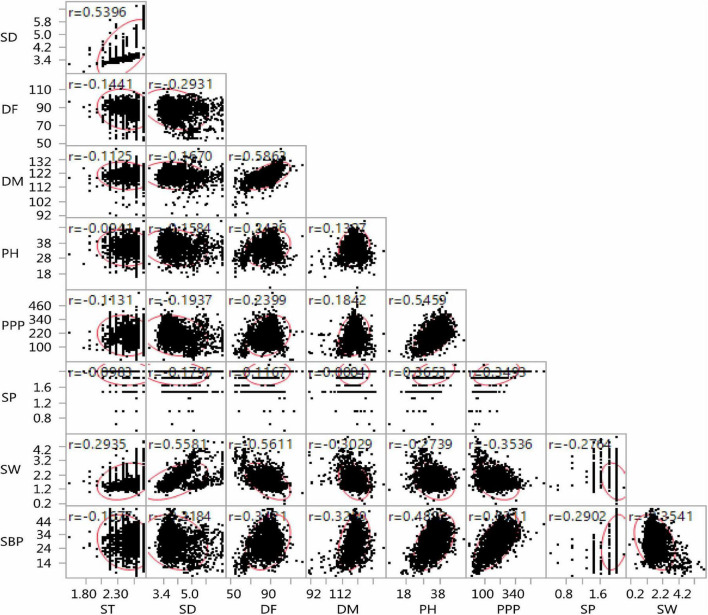
Scatter plot matrix showing the correlation between nine agro-morphological traits of the entire lentil germplasm conserved in the Indian national genebank.

**FIGURE 10:**
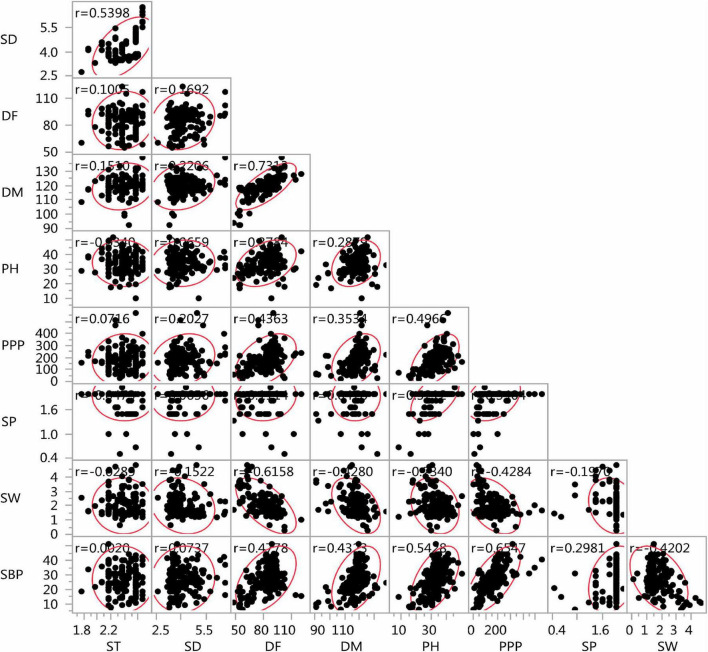
Scatter plot matrix showing the correlation between nine agro-morphological traits of the Indian national genebank lentil core set.

The lentil germplasm accessions recorded significant and positive correlations between seed thickness and seed diameter, equally in both entire set and core set (*r* = 0.539) and between days to 50% flowering and days to 80% maturity (*r* = 0.582 in the entire set, *r* = 0.731 in the core set). Plant height was positively correlated with days to 50% flowering (*i* = 0.378), days to 80% maturity (*r* = 0.287), pods per plant (*r* = 0.496), seeds per pod (*r* = 0.521), and number of secondary branches (*r* = 0.542) and negatively with seed thickness (*r* = –0.034) and seed diameter (*r* = –0.234) in the core set. Similar trends were recorded in the entire lentil collection. The number of secondary branches was positively correlated with days to 50% flowering, days to 80% maturity, plant height, pods per plant, and seeds per pod negatively with 100-seed weight, both in the core collection and entire set (Figures 9, 10).

Principal component analysis revealed the association among different descriptors and their contribution toward variability. The first three PCA components provided a realistic summary of the data and explained 70.69% of the total variation in the core set. The first principal component (PC1) accounted for 39.36% of the total variation, whereas PC2 and PC3 accounted for 17.73% and 13.60% variance, respectively. These values were comparable with those of the entire lentil collection, explaining 68.03% of the variation (PC1:37.49%; PC2:16.62%, and PC3:13.91%). The negative variation in PC1 was mainly contributed (>50%) by days to 50% flowering, days to 80% maturity, plant height, number of secondary branches per plant, and pods per plant and 100-seed weight in the entire and lentil core set (Supplementary Table 4 and Supplementary Figure 1).

## Discussion

**FIGURE 11:**
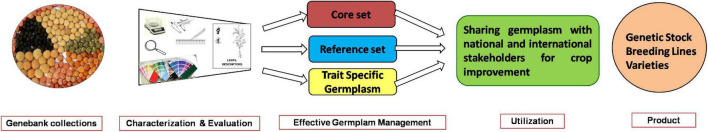
Flowchart depicting the stepwise utilization of Indian national genebank lentil collections.

Genebanks are reservoirs of germplasm variability containing vital genes. These play a crucial role in crop-breeding programs (Qiu et al., 2013; Sandhu and Singh, 2021). Efficient and effective use of genebank collections in crop improvement depends on a thorough understanding of the existing genetic variability, knowledge of the genes present in individual accessions, and their value for use (Ortiz, 2002). Characterization and evaluation are the most crucial activities of plant genetic resources (PGR) management (Jones, 1984). Its scope has increased due to the increasing emphasis on conservation. The use of genebank accessions through characterization and evaluation has resulted in the development of genetic stocks, breeding lines, and commercial varieties (Figure 11; Carvalho et al., 2013).

### Agro-Morphological Characterization of Lentil Germplasm

Geo-referenced maps of India revealed that most of the Indian accessions were from the Gangetic plains and eastern part of India belonging to Uttar Pradesh (254), Bihar (203), Uttarakhand (181), and Madhya Pradesh (96). These are the most populated regions of India, with the massive scope of pulse production in their rice fallow areas (Chowdhury et al., 2020). The majority of the germplasm was collected from hot subhumid (dry and moist) to hot subhumid agro-ecological zones of India. In contrast, the ECs, conserved in INGB, represented all six continents, primarily Asia.

The major steps in crop improvement are assessing the variability for desired traits and their utilization in breeding programs (Kumar et al., 2015). Previous researchers adopted the piecemeal approach to characterize the agro-morphological traits in lentil germplasm collections (Gautam et al., 2013; Jha et al., 2014; Kumar and Solanki, 2014). However, in this study, the entire genebank collections representing all agro-ecological zones were characterized in one go. Significant differences among lentil accessions based on agro-morphological characterization indicated their potential for use in current and future lentil breeding programs. The results showed significant variations in certain traits such as days to flowering, seed thickness, days to maturity, number of secondary branches, 100-seed weight, number of pods per plant, and seed diameter. The wide range of variability obtained was attributable to the diverse collection assessed, involving native and introduced germplasm originating from diverse geographical origins with different genetic makeup (Phogat et al., 2020).

Lentil is a less vigorous grain legume that faces competition from weeds during the early growth stage. Lentil germplasm with increased vigor can dominate the weeds, ultimately enhancing the yield (Sharma et al., 2018). The previous researchers reported that EPV and rapid canopy development as key traits for escaping drought at the terminal stage because these traits lead to the early onset of maturity (Sarker et al., 2005; Kumar et al., 2012). The majority of exotic accessions had light-green leaves, whereas indigenous varieties were dominated by green to dark green canopy. In addition, the purple pigmentation was observed to be disappeared with the increase in the ambient temperature. Similarly, in subterranean clover, Nichols et al. (1996) reported prominence of purple pigmentation during winter, which is appeared with the arrival of spring. Wide variations were recorded in leaf pubescence, ranging from the absence of pubescence to dense pubescence. Low or moderate leaf pubescence has promoted tolerance to aphids (Kumari et al., 2009). However, Tripathi et al. (2020) indicated that pubescence on the pod is a unique feature of cowpea, a marker-trait for insect resistance. Variation was also reported in leaflet size, tendril length, flower ground color, pod shedding, pod dehiscence, testa color, and seed shape.

Elias et al. (1979) suggested that the protein quality is affected by seed coat color in beans. Diversity in seed color among accessions may provide valuable genetic resources for biofortified lentils. Orange cotyledons are preferred by Indians, which is also reflected in Indian genebank collections (Singh et al., 2014). These agro-morphological descriptors can facilitate the differentiation of distinct phenotypic classes and are used as diagnostic keys for taxonomic delineation (Pundir et al., 1985; Gore et al., 2019). The mode of inheritance of these traits can be investigated using the principles of classical genetics. This mega characterization program showed that most of the Mediterranean germplasm were found with low biomass and poor yield. The direct use of these accessions appeared difficult in Indian conditions, leading to the ubiquitous selection of Indian germplasm with high biomass and better yield traits.

The entire lentil germplasm was more diverse for quantitative traits such as number of secondary branches per plant, pods per plant, plant height, days to 50% flowering, seed thickness and qualitative traits, leaf pubescence, biomass score, pod shedding, growth habit, leaflet size, and pod dehiscence. Plant height (cm) in the INGB lentil core set ranged from 9.70 to 52.13 with mean value of 34.04, while it was ranged from 15 to 40 cm with mean value of 23 cm in the USDA core set sown at Pullman, Washington, United States, in 1996 (Tullu et al., 2001). An average value of days to flowering and maturity is lower in the USDA lentil core set than the INGB lentil core set (Tullu et al., 2001). The grouping of germplasm in different clusters allowed us to study their genetic diversity, whereas the heatmap depicted the trait diversity. Germplasm accessions belonging to diverse clusters can be used in cross-breeding programs and recombination studies; for example, Clusters XI comprised early maturing accessions such as IC241529, IC241531, and IC241532 (less than 100 days), and cluster X comprised late maturing accessions such as EC267554, EC267615, IC258265, and EC440747 > 125 days. Similarly, Clusters XI had accessions with low plant height IC241488, IC241501, IC241529, and IC241531 (<20 cm) and cluster V had accessions with larger plant height, IC329110, IC398793, and IC59038 (>50 cm). The accessions such as EC223214, IC241543 (with high 100-seed weight) grouped in cluster XII and may be crossed with accessions with low seed weight IC361467, IC521438 (<1.4 g) from cluster III for genetics and mapping purposes.

### Identification of Trait-Specific Germplasm

The ultimate aim of germplasm characterization is to support crop-breeding programs (He and Li, 2020; Guerra-García et al., 2021). Similarly, the present work unfolded the treasure of lentil collections conserved in the NGB. The accession IC317520 collected from Rajasthan was identified as a unique seed morphotype with intact funiculus at maturity (Tripathi et al., 2019). This was reported for the first time and is registered as a genetic stock (INGR19072) for its utilization in lentil breeding programs. An Intact funiculus may provide resilience to drought stress, resulting in high yields. This trait may help to gain area in rice-fallow areas of eastern India, where the crop is sown at conditions of conserved moisture (Lamichaney et al., 2021; Tripathi et al., 2021). Rice–wheat and rice–rice systems in the Indo-Gangetic plains dominate South Asia’s cereal systems (Kumar et al., 2013). A substantial reduction in the acreage under pulses is attributed to the expansion of high-yielding wheat and boro rice in northern India and eastern to north-eastern India, respectively. This trend of pushing out pulses is likely to continue unless extra-early varieties that fit into these cropping systems are developed (Erskine et al., 1993; Shrestha et al., 2006). Similarly, in the rice-fallow areas of South Asia, the top soil layer generally dries at the harvesting stage, making it impossible to sow the next crop. Under such conditions, extra-early lentil germplasm can be used as donors to breed early maturing varieties to fit into the cropping system and convert these mono-cropped areas into double-cropped areas, thereby increasing the production of lentils under rice-based systems. In a previous study, a lentil accession with 59 days to flower was reported (Kumar and Solanki, 2014). However, the present experiment discovered accessions, IC241529 and IC241532, flowered in 51 days. The number of pods per plant and the number of secondary branches are the primary quantitative descriptor traits that can be used as kickstarting points for selecting high-yielding germplasm. Further, the seed size and shape of lentils are important traits because they affect the market class, cooking time, and the quality and yield of milled lentils (Fedoruk, 2013). Indigenous and exotic accessions are broadly classified into small- and large-seeded types, respectively. Descriptor traits, such as tall, erect, top pod-bearing habits, thick stems, synchronous maturity, lodging tolerance, and non-shattering reported contributing to the development of varieties with machine harvestability (Kumar et al., 2013). Genetic variability for these traits was recorded in the INGB lentil germplasm. In an earlier study, Idleb-2 variety was released for machine harvesting (El-Ashkar et al., 2004). Identified germplasm, EC267615, may provide avenues for breeding machine harvestable varieties in India. Multiflowering accessions can be identified with the expression of three or more flowers at one or multiple flowering nodes (Gaur and Gour, 2002; Benlloch et al., 2015; Sanwal et al., 2016; Devi et al., 2018). In several legumes, previous researchers have documented multiflowering clusters for a few flowering nodes in *Cicer* (up to nine FPP) (Gaur and Gour, 2002), *Pisum* (up to five FPP) (Devi et al., 2018), and *Lens* (up to seven FPP) (Mishra et al., 2020). However, this study found a unique multiflowering germplasm accession, IC241473, in cultivated lentils, formed up to 16 FPP at multiple flowering nodes. This trait can fulfill the aim of genebanks to work in the direction of conservation and utilization. Trait specific germplasm (TSG) developed in this study were shared with the National Agricultural Research System (NARS) partners under the material transfer agreement for utilization in the varietal development programs.

### Development of Core Collection

Globally, the first and foremost reason behind the limited use of conserved germplasm by plant breeders is huge and uncharacterized germplasm collections in certain genebanks (Ortiz et al., 2008). Characterized germplasm and smaller subsets can enhance germplasm use in trait discovery (Dutta et al., 2015). Core sets are subsets of large and diffused germplasm collections, containing selected accessions that represent the genetic variability of the entire set and acts as the entry point to the entire collection for trait-specific evaluation (Ortiz et al., 1998, 1999; Huamán et al., 1999; Upadhyaya et al., 2003). PowerCore is a fast and reliable tool for extracting a core set with a higher percentage of diversity than other methods used (Kim et al., 2007; Zhang et al., 2011; Dutta et al., 2015).

A subsampling procedure based on 26 agro-morphological descriptors and geographical origin data resulted in the selection of 170 germplasm accessions as the lentil core set, whereas only 79 accessions were sampled without passport information. Therefore, the inclusion of stratification into the geographical origin and places should be a choice for the sampling procedure. The INGB lentil core set amounted to 7.31% of the entire collection (2,324 accessions) conserved in the INGB, New Delhi. Similarly, Archak et al. (2016) selected 1,103 accessions (7.5%) as the chickpea core set from 14,651 accessions conserved in the INGB. As expected, the INGB lentil core set comprised 80.58% accessions belonging to Indian origin. However, only 12.89% (37 accessions) of the Indian accessions contributed to a lentil core set developed for agro-morphological descriptor traits by United States Department of Agriculture (USDA) (Tullu et al., 2001). The USDA lentil core consists of 12% of the 2,390 lentils (Simon and Hannan, 1995; Tullu et al., 2001). The maximum representation of germplasm accessions in INGB lentil core collections was from India (137), followed by Syria (22), indicating the rich diversity of cultivated lentils in India. A total of 95.88% of the core set belonged to Asian countries, including 15.29% from other Asian countries (Syria [mainly from ICARDA], Bangladesh, Israel, and Pakistan). As lentil is the main crop of Asia (Laskar et al., 2019), the INGB core set can be used as a diversity hub to meet the present and future challenges of the Asian lentil breeding program. Simon and Hannan (1995) also reported the utility of the legume cores in chickpea, lentil, and pea developed by USDA for directing users toward desirable germplasm from targeted geographic regions, and facilitating users at the preliminary stages of germplasm evaluation.

While sampling for a core set, the phenotypic associations in the core set need to be preserved for maintaining coadapted genetic complexes (Ortiz et al., 1998) and efficient utilization of germplasm (Upadhyaya et al., 2001). Nine traits were studied and 36 comparisons were performed between the pairs of characteristics. All trait combinations maintained similarity in correlation coefficient value between the entire INGB collection and the INGB core set. Earlier studies on the development of core in various crops such as chickpea (Upadhyaya et al., 2001; Archak et al., 2016), eggplant (Gangopadhyay et al., 2010), and wheat (Phogat et al., 2020) also reported the preservation of trait association in the core set. The strong correlation among some of the traits, such as days to 50% flowering and days to maturity (*r* = 0.52 in the entire collection, *r* = 0.731 in core set), and secondary branches per plant and pods per plant (*r* = 0.63 in the entire collection, *r* = 0.65 in core set), showed that future germplasm characterization might use days to 50% flowering and secondary branches per plant during preliminary evaluation. This trait is easier to measure than other traits, such as days to maturity and pods per plant. Similar observations were reported by Upadhyaya et al. (2003). Examination of the entries’ spatial distribution and explaining the variance through PCA were also reported as an exploratory criterion for evaluating the core set in the study of Bisht et al. (1998) and Wang et al. (2008). In the present study, the first three PCs collectively explained 70.39% of the total variation in the core sets and 68.03% in the entire set. Germplasm accessions were majorly distributed in one large group and one small group in both the complete set and the core collection of INGB lentil germplasm.

## Conclusion

The present study was based on the characterization of 2,324 accessions of cultivated lentils that resulted in the development of a core set of 170 accessions. The core set possessed a high diversity and allelic richness for major descriptor traits as revealed by the summary statistics, chi-square test, and SDI. The lentil core set described in this study represents the variability existing in the germplasm conserved in the INGB and provides insights into the traits for earliness, seed size, growth habit, and other vital agronomic traits. Identified and validated TSGs could serve as a potential source of novel alleles and genes. Characterization data of INGB lentil germplasm and developed multipurpose core set may serve as a valuable resource for lentil workers and open the avenues for germplasm utilization in the selection, mapping, genomics, and trait discovery to attain sustainable lentil production under climate changing regime.

## Data Availability Statement

The original contributions presented in the study are included in the article/Supplementary Material, further inquiries can be directed to the corresponding author/s.

## Author Contributions

KT and AK planned and designed the research. KT, PG, AKS, GC, RBh, RBa, and RM assisted in data recording. JK, KT, and DM analyzed the data. NS, VG, and PG contributed to the materials. DS and PG performed the passport data analysis and geo-referenced map. KT and PG prepared the manuscript. AS, GM, JR, and HD edited the manuscript. KS provided the necessary support for inter-institutional collaboration. All the authors contributed to the article and approved the submitted version.

## Conflict of Interest

The authors declare that the research was conducted in the absence of any commercial or financial relationships that could be construed as a potential conflict of interest.

## Publisher’s Note

All claims expressed in this article are solely those of the authors and do not necessarily represent those of their affiliated organizations, or those of the publisher, the editors and the reviewers. Any product that may be evaluated in this article, or claim that may be made by its manufacturer, is not guaranteed or endorsed by the publisher.
