# Supplementary material for: Agro-Morphological Characterization of Lentil Germplasm of Indian National Genebank and Development of a Core Set for Efficient Utilization in Lentil Improvement Programs
Source: Front Plant Sci. 2022 Jan 27;12:751429. doi: 10.3389/fpls.2021.751429 (PMC8828943; doi:10.3389/fpls.2021.751429)
Supplement: Supplementary file 1 [file Table_1.DOCX]

**Table S1. ANOVA and descriptive statistics of entire lentil germplasm grown over two years.**

| **Traits** | **Minimum** | **Maximum** | **Mean** | **Std. Error** | **CV(%)** | **Skewness** | **Kurtosis** | **F Statistics (Treatment)** | **F Statistics (Year)** |
| --- | --- | --- | --- | --- | --- | --- | --- | --- | --- |
| **ST** | 1.60 | 2.70 | 2.42 | .01 | 6.96 | 1.671 | 2.892 | 1.16** | 32.18 |
| **SD** | 2.73 | 6.80 | 4.18 | .02 | 17.41 | 1.734 | 3.276 | 1.08** | 63.89 |
| **DF** | 51.00 | 123.00 | 88.96 | .188 | 10.17 | -1.148 | 1.582 | 5.58** | 3928.99** |
| **DM** | 93.00 | 140.00 | 120.88 | .083 | 3.28 | -1.193 | 7.152 | 2.07** | 3842.46** |
| **PH** | 9.70 | 52.13 | 35.34 | .100 | 13.64 | -.516 | .805 | 2.00** | 33.10** |
| **PP** | 20.83 | 571.33 | 206.38 | 1.483 | 34.56 | .078 | .434 | 1.85** | 649.78** |
| **SP** | 1.00 | 2.00 | 1.97 | .003 | 6.38 | -5.094 | 32.724 | 1.13** | 33.31 |
| **SW** | 0.75 | 7.6 | 1.81 | .011 | 28.50 | 1.811 | 4.521 | 4.90** | 698.04** |
| **SBP** | 6.00 | 51.50 | 28.67 | .163 | 27.33 | -.300 | .029 | 2.44** | 3152.56** |

**Table S2.Cluster-wise mean values for traits studied in lentil germplasm**

| **Cluster** | **ST** | **SD** | **DF** | **DM** | **PH** | **PPP** | **SP** | **SW** | **SBP** |
| --- | --- | --- | --- | --- | --- | --- | --- | --- | --- |
| 1 | 2.281 | 3.974 | 94.786 | 123.765 | 31.668 | 162.661 | 1.997 | 1.685 | 25.976 |
| **2** | 2.251 | 3.814 | 91.894 | 121.508 | 37.157 | 219.532 | 1.995 | 1.589 | 31.897 |
| 3 | 2.488 | 3.677 | 92.221 | 120.283 | 34.816 | 189.601 | 1.998 | 1.523 | 26.171 |
| 4 | 2.517 | 3.663 | 92.160 | 121.812 | 38.582 | 250.942 | 1.998 | 1.538 | 34.864 |
| 5 | 2.293 | 3.907 | 90.399 | 121.555 | **39.391** | **305.808** | 1.993 | 1.673 | **38.316** |
| 6 | 2.492 | 4.605 | 76.413 | 115.128 | 32.025 | 150.524 | 1.996 | 2.472 | 21.572 |
| 7 | 2.350 | 4.059 | 78.086 | 118.184 | 34.952 | 207.268 | 1.997 | 1.941 | 25.773 |
| 8 | **2.647** | **5.686** | 89.301 | 120.418 | 36.587 | 224.736 | 1.999 | 1.790 | 29.231 |
| 9 | 2.488 | 4.587 | 88.695 | 121.150 | 37.774 | 218.633 | **2.000** | 2.369 | 33.075 |
| 10 | 2.446 | 4.406 | 93.023 | 124.315 | 28.658 | 112.692 | 1.459 | 2.028 | 21.711 |
| **11** | 2.610 | 5.311 | 68.961 | **113.039** | 27.383 | 78.605 | 1.539 | 3.197 | 14.276 |
| 12 | 2.604 | 5.233 | **67.923** | 115.931 | 28.818 | 82.749 | 1.910 | **3.239** | 15.167 |

*Whereas ST: Seed Thickness; SD: Seed Diameter; DF: Days to 50% Flowering; DM: Days to 80% Maturity; PH: Plant Height; PPP: Pods Per Plant; SP: Seeds Per Pod; SW: 100-Seeds Weight and SBP: Number of Secondary Branches per plant

**Table S3. List of Indian national genebank lentil core set**

| **S.N.** | **Accession** | **Collection source/ developer institution /country** | **Cluster*** |
| --- | --- | --- | --- |
| **1** | EC241480 | Israel | I |
| **2** | EC267362 | ICARDA, Syria | XI |
| **3** | EC223241 | ICARDA, Syria | XII |
| **4** | EC223243 | ICARDA, Syria | XI |
| **5** | EC397812 | Kova Research Instt of Plant Production, Slovakia | I |
| **6** | EC223237 | ICARDA, Syria | XII |
| **7** | EC225503 | ICARDA, Syria | XII |
| **8** | EC223235 | ICARDA, Syria | XII |
| **9** | IC584611 | India | VI |
| **10** | IC241507 | India | XII |
| **11** | IC145259 | Uttar Pradesh, India | IV |
| **12** | IC240886 | India | II |
| **13** | IC201798 | Uttar Pradesh, India | VII |
| **14** | IC14158B | Uttar Pradesh, India | III |
| **15** | IC560225 | Delhi | I |
| **16** | EC440870 | ICARDA, Syria | I |
| **17** | IC392561 | Uttarakhand, India | X |
| **18** | IC240885 | India | IV |
| **19** | IC614705 | Madhya Pradesh, India | III |
| **20** | IC553069 | Uttarakhand, India | II |
| **21** | IC201568 | Punjab, India | II |
| **22** | IC258265 | Himachal Pradesh, India | X |
| **23** | IC145305 | Uttarakhand, India | V |
| **24** | IC381285 | Himachal Pradesh, India | I |
| **25** | EC440747 | ICARDA, Syria | X |
| **26** | IC16453 | Maharashtra, India | V |
| **27** | IC201533 | Uttar Pradesh, India | IV |
| **28** | IC98401 | Himachal Pradesh, India | II |
| **29** | IC53237 | Chattisgarh, India | II |
| **30** | IC59038 | Bihar, India | V |
| **31** | IC381127 | Himachal Pradesh, India | I |
| **32** | IC201564 | Punjab, India | II |
| **33** | EC441573 | ICARDA, Syria | I |
| **34** | IC384445 | Madhya Pradesh, India | X |
| **35** | EC955432 | Bangladesh | X |
| **36** | IC241444 | India | I |
| **37** | EC225504 | ICARDA, Syria | XI |
| **38** | IC384473 | Madhya Pradesh, India | VI |
| **39** | IC241531 | India | XI |
| **40** | IC78387 | India | V |
| **41** | EC223239 | ICARDA, Syria | V |
| **42** | IC520809 | Jharkhand, India | IV |
| **43** | IC421976 | Himachal Pradesh, India | IX |
| **44** | IC417864 | Bihar, India | IV |
| **45** | IC78529 | India | VIII |
| **46** | IC321501 | Bihar, India | IV |
| **47** | IC361417 | Bihar, India | II |
| **48** | IC260965 | Uttarakhand, India | VIII |
| **49** | EC329161 | USDA. ARSS Grain Legumes Genetes and physio Logy Res-215 Johnson Hall WSU, Pullman WA 99164 U.S.A. | X |
| **50** | IC274090 | Madhya Pradesh, India | VI |
| **51** | IC521438 | Jharkhand, India | III |
| **52** | IC78455 | India | I |
| **53** | IC277173 | India | III |
| **54** | IC544563 | Bihar, India | II |
| **55** | IC73121 | Odisha, India | VI |
| **56** | IC95654 | Andaman and Nicobar Islands, India | II |
| **57** | IC78549 | India | I |
| **58** | IC398793 | Bihar, India | V |
| **59** | IC201582 | Maharashtra, India | VII |
| **60** | IC98392 | Himachal Pradesh, India | IV |
| **61** | IC385824 | Jharkhand, India | X |
| **62** | IC201557 | Punjab, India | VI |
| **63** | IC241533 | India | VI |
| **64** | IC95658 | Jharkhand, India | II |
| **65** | IC199779 | India | II |
| **66** | IC208326 | Uttar Pradesh, India | VII |
| **67** | IC260062 | Bihar, India | III |
| **68** | EC33920 | CSIRO , Canberrra , Australia | I |
| **69** | IC201788 | Uttar Pradesh, India | III |
| **70** | IC14726 | Assam, India | I |
| **71** | IC260010 | Odisha, India | VII |
| **72** | IC33920 | Maharashtra, India | I |
| **73** | IC11871 | Maharashtra, India | I |
| **74** | IC241222 | India | VI |
| **75** | IC396044 | Chattisgarh, India | VI |
| **76** | IC201661 | Uttar Pradesh, India | I |
| **77** | IC201776 | Uttar Pradesh, India | IV |
| **78** | IC201693 | Uttar Pradesh, India | VII |
| **79** | IC201656 | Uttar Pradesh, India | I |
| **80** | IC98364 | Bihar, India | III |
| **81** | IC201697 | Telangana, India | I |
| **82** | IC361467 | Bihar, India | III |
| **83** | EC16391 | Jardin Botanique, de la ville de Geneve 192, Route de Lausanne, Geneve - suisse, Switzerland | III |
| **84** | IC329109 | Madhya Pradesh, India | IX |
| **85** | IC396758 | Madhya Pradesh, India | VIII |
| **86** | IC241475 | India | XII |
| **87** | IC241501 | India | XI |
| **88** | IC22651 | Chattisgarh, India | V |
| **89** | IC267088 | Uttar Pradesh, India | VI |
| **90** | IC267656 | Bihar, India | VIII |
| **91** | IC139850 | Madhya Pradesh, India | V |
| **92** | IC201555 | Haryana, India | V |
| **93** | EC28514 | Chief Div. Of Plant & Seed Control , Agric. Bldg., Private Bag 179, Pretoria, South Africa | VII |
| **94** | IC22658 | Chattisgarh, India | III |
| **95** | EC223212 | ICARDA, Syria | XI |
| **96** | IC241454 | India | VI |
| **97** | IC241144 | India | VIII |
| **98** | EC267554 | ICARDA, Syria | X |
| **99** | IC201748 | Uttar Pradesh, India | II |
| **100** | IC241543 | India | XII |
| **101** | IC60969 | Jammu and Kashmir, India | XII |
| **102** | IC98391 | Himachal Pradesh, India | IV |
| **103** | IC447768 | Jharkhand, India | IV |
| **104** | IC201781 | Uttar Pradesh, India | III |
| **105** | IC345433 | Maharashtra, India | III |
| **106** | IC201710 | Uttarakhand, India | I |
| **107** | IC316127 | Uttar Pradesh, India | IX |
| **108** | EC223238 | ICARDA, Syria | XI |
| **109** | IC342718 | Bihar, India | IX |
| **110** | IC283540 | Bihar, India | III |
| **111** | IC201772 | Uttar Pradesh, India | V |
| **112** | IC280887 | Assam, India | VII |
| **113** | IC201553 | Rajasthan, India | IV |
| **114** | IC241488 | India | XI |
| **115** | EC223214 | ICARDA, Syria | XII |
| **116** | IC201684 | Haryana, India | II |
| **117** | EC373712 | ICARDA, Syria | III |
| **118** | IC201558 | Punjab, India | IV |
| **119** | IC329110 | Madhya Pradesh, India | V |
| **120** | EC299645 | ICARDA , Syria | I |
| **121** | EC299650 | ICARDA , Syria | VIII |
| **122** | IC345478 | Gadkumbali,,Bhandara,Maharashtra | VII |
| **123** | IC201537 | Uttar Pradesh, India | IV |
| **124** | EC329164 | USDA. ARSS Grain Legumes Genetes and physio Logy Res-215 Johnson Hall WSU, Pullman WA 99164 U.S.A. | IV |
| **125** | IC267668 | Bihar, India | II |
| **126** | IC384447 | Madhya Pradesh, India | IX |
| **127** | IC53238 | Chattisgarh, India | III |
| **128** | IC78398 | India | V |
| **129** | IC424864 | Bihar, India | IV |
| **130** | IC201548 | Uttar Pradesh, India | IV |
| **131** | IC346268 | Bihar, India | I |
| **132** | IC267105 | Uttar Pradesh, India | VII |
| **133** | IC78406 | India | V |
| **134** | EC955431 | Bangladesh | V |
| **135** | IC616579 | New Delhi,Delhi | IX |
| **136** | IC201694 | Uttar Pradesh, India | VII |
| **137** | IC201562 | Punjab, India | IV |
| **138** | IC565035 | West Bengal, India | IX |
| **139** | EC299732 | ICARDA , Syria | VIII |
| **140** | IC241447 | India | VI |
| **141** | IC405259 | Haryana, India | IX |
| **142** | IC201561 | Punjab, India | I |
| **143** | IC78477 | India | VI |
| **144** | IC267078 | Uttar Pradesh, India | VIII |
| **145** | IC148333 | Uttar Pradesh, India | V |
| **146** | IC541007 | Uttar Pradesh, India | IX |
| **147** | EC27659 | Pakistan | VI |
| **148** | IC241529 | India | XI |
| **149** | IC316132 | Uttarakhand, India | II |
| **150** | IC22666 | Madhya Pradesh, India | III |
| **151** | IC241532 | India | VI |
| **152** | EC267615 | ICARDA,Syria | X |
| **153** | IC199461 | Uttar Pradesh, India | II |
| **154** | EC267595 | ICARDA,Syria | I |
| **155** | IC317520 | Rajasthan, India | I |
| **156** | IC241473 | India | VIII |
| **157** | EC499760 | United States of America | XII |
| **158** | IC78552 | Andaman and Nicobar Islands, India | II |
| **159** | EC267578 | ICARDA,Syria | II |
| **160** | IC560153 | Delhi, India | II |
| **161** | IC560177 | Delhi, India | X |
| **162** | IC469404 | Uttar Pradesh, India | VII |
| **163** | IC593588 | Uttar Pradesh, India | VII |
| **164** | IC612197 | Uttar Pradesh, India | VI |
| **165** | JL-3 | Madhya Pradeash, India | VI |
| **166** | IC560159 | Delhi, India | VII |
| **167** | IC560160 | Delhi, India | VII |
| **168** | IC241240 | India | VI |
| **169** | IC574485 | Punjab , India | VII |
| **170** | IC540405 | Delhi, India | VII |

*****Number represents the cluster of **dendrogram generated by performing hierarchical cluster analysis.**

**Table S4. Eigenvectors and eigenvalues for entire collection and core-set of lentil germplasm.**

|  |  |  |  | **Factor loadings (Entire set)** | | | | | | | | |
| --- | --- | --- | --- | --- | --- | --- | --- | --- | --- | --- | --- | --- |
|  | **Eigenvalue** | **% Variance** | **Cumulative** | **ST** | **SD** | **DF** | **DM** | **PH** | **PPP** | **SP** | **SW** | **SBP** |
| **PC1** | 3.374 | 37.492 | 37.492 | -0.418 | -0.587 | 0.660 | 0.507 | 0.618 | 0.689 | 0.467 | -0.747 | 0.727 |
| **PC2** | 1.497 | 16.628 | 54.121 | 0.478 | 0.499 | -0.311 | -0.292 | 0.502 | 0.481 | 0.390 | 0.297 | 0.336 |
| **PC3** | 1.252 | 13.911 | 68.031 | 0.522 | 0.437 | 0.488 | 0.643 | -0.060 | -0.031 | -0.343 | 0.047 | 0.110 |
|  |  |  |  | **Factor loadings (**Core^d^ ) | | | | | | | | |
| **PC1** | 3.543 | 39.365 | 39.365 | 0.149 | 0.299 | 0.791 | 0.701 | 0.673 | 0.768 | 0.438 | -0.684 | 0.782 |
| **PC2** | 1.596 | 17.730 | 57.094 | 0.787 | 0.719 | 0.157 | 0.266 | -0.365 | -0.119 | -0.408 | -0.060 | -0.215 |
| **PC3** | 1.224 | 13.602 | 70.697 | 0.367 | 0.408 | -0.415 | -0.400 | 0.344 | 0.179 | 0.584 | 0.313 | 0.028 |

**^
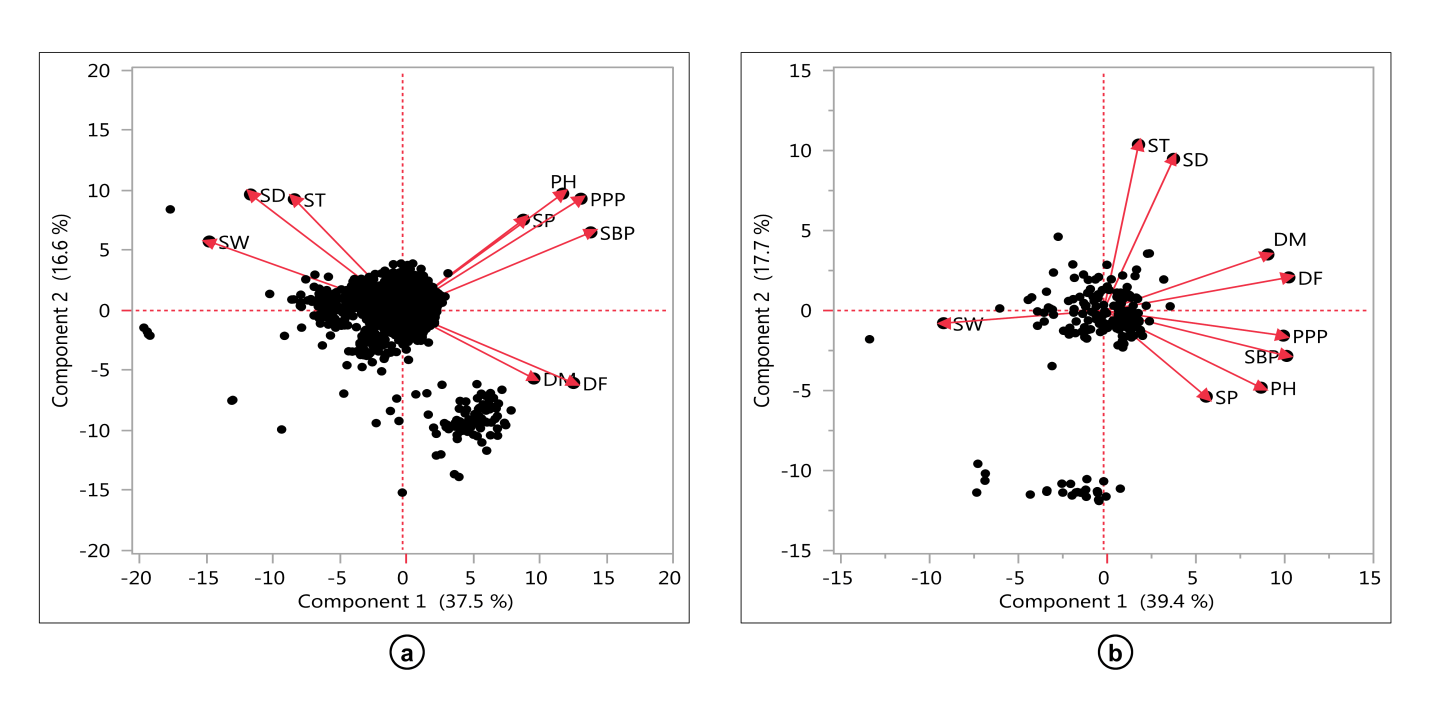
^**

**Figure S1. Principal component biplot; a) entire collections of INGBb) INGB core set**
